# Factors influencing dietary practices in a transitioning food environment: a cross-sectional exploration of four dietary typologies among rural and urban Ugandan women using Photovoice

**DOI:** 10.1186/s12937-020-00634-9

**Published:** 2020-11-25

**Authors:** Carolyn I. Auma, Rebecca Pradeilles, Megan K. Blake, David Musoke, Michelle Holdsworth

**Affiliations:** 1 School of Health and Related Research, University of Sheffield, 30 Regent Street, Sheffield, S1 4DA UK; 2 School of Sport, Exercise and Health Sciences, Loughborough University, Sheffield, LE11 3TU UK; 3 Department of Geography, University of Sheffield, Winter Street, Sheffield, S3 7ND UK; 4 School of Public Health, Makerere University, New Mulago Hill Road, Kampala, Uganda; 5 UMR NUTRIPASS: Institute of Research for Development- IRDUM, SupAgro Montpellier, Montpellier, France

**Keywords:** Dietary practices, Dietary clusters, Dietary typologies, Photovoice, Environmental sustainability, Factors, Women, Uganda

## Abstract

**Background:**

Healthy and sustainable dietary practices offer a possible solution to competing tensions between health and environmental sustainability, particularly as global food systems transition. To encourage such dietary practices, it is imperative to understand existing dietary practices and factors influencing these dietary practices. The aim of this study was to identify multi-level factors in lived rural and urban Ugandan food environments that influence existing dietary practices among women of reproductive age (WRA).

**Methods:**

A mixed methods study was conducted. Multiple correspondence analysis followed by hierarchical cluster analysis were performed on dietary data collected among a sample (*n* = 73) of Ugandan WRA in Kampala (urban) and Wakiso (rural) districts to elicit dietary clusters. Dietary clusters, which were labelled as dietary typologies based on environmental impact and nutrition transition considerations, were reflective of dietary practices. Following this, a smaller sample of WRA (*n* = 18) participated in a Photovoice exercise and in-depth interviews to identify factors in their social, physical, socio-cultural and macro-level environments influencing their enactment of the identified dietary typologies, and therefore dietary practices.

**Results:**

Four dietary typologies emerged: ‘*urban, low-impact, early-stage transitioners*’, ‘*urban, medium-impact, mid-stage transitioners*’, ‘*rural, low-impact, early-stage transitioners*’ and ‘*rural, low-impact, traditionalists*’. Although experienced somewhat differently, the physical environment (access, availability and cost), social networks (parents, other family members and friends) and socio-cultural environment (dietary norms) were cross-cutting influences among both urban and rural dietary typologies. Seasonality (macro-environment) directly influenced consumption of healthier and lower environmental impact, plant-based foods among the two rural dietary typology participants, while seasonality and transportation intersected to influence consumption of healthier and lower environmental impact, plant-based foods among participants in the two urban dietary typologies.

**Conclusion:**

Participants displayed a range of dietary typologies, and therefore dietary practices. Family provides an avenue through which interventions aimed at encouraging healthier and lower environmental impact dietary practices can be targeted. Home gardens, urban farming and improved transportation could address challenges in availability and access to healthier, lower environmental impact plant-based foods among urban WRA.

## Background

By 2050, approximately 70% of people worldwide will reside in urban areas [1]. Most urbanisation is expected to take place in low- and middle-income countries (LMICs) in Africa and Asia. Indeed, between 2018 and 2050, together India, China and Nigeria are expected to account for 35% of global urban growth [1]. As countries urbanise, food systems transition, concomitantly resulting in changes in food environments to which people are exposed [2]. Consequently, this could result in shifts in dietary patterns and practices from the more ‘traditional’ (mostly plant-based and less processed) towards more ‘westernised’ diets (high in saturated and trans fats, refined carbohydrates, sugars, animal protein and ultra-processed foods) [2, 3]. This is what is commonly referred to as the nutrition transition [3]. Transitions in food systems and food environments, and consequently dietary patterns and practices, have implications for both health and environmental sustainability [4–6].

On the one hand, dietary transitions could result in increased dietary diversity, which could have positive benefits for nutritionally vulnerable sub-groups, e.g. WRA and adolescent girls, by increasing intakes of micronutrients that are usually deficient, e.g. zinc, vitamin B12 and iron [7, 8]. However, a significant body of literature, mainly from high-income countries (HICs), has demonstrated an association between so-called ‘westernised’ dietary patterns and overweight, obesity and nutrition-related non-communicable diseases (NR-NCDs) like type 2 diabetes [3, 5]. Moreover, unlike what has been previously observed in HICs, in LMICs, which usually have strained public health systems, these NR-NCDs are increasingly prevalent among the poor, putting them at risk of economic stress incurred in addressing chronic healthcare needs [9]. Further to this, literature suggests that in LMIC contexts dietary changes are first seen in urban areas compared with rural areas. Moreover, in these contexts, younger (25–44 years), lower-income women are particularly vulnerable to overweight and obesity, compared with men of the same age group and older women [10–12]. In addition to negative health outcomes, so called ‘westernised’ dietary patterns have been demonstrated to have negative implications for environmental sustainability [5, 6, 13]. Recent literature suggests that these dietary patterns are associated with both higher water footprint and greenhouse gas emission (by weight) owing to high consumption of ruminant meat (beef, mutton and pork), dairy, poultry and fish at the expense of plant-based foods, such as fresh roots and tubers, nuts and seeds, pulses, fruit and vegetables [5, 6, 14].

Healthy and environmentally sustainable dietary patterns and practices have been highlighted as a possible solution to address both health and environmental sustainability concerns as food systems transition globally [13]. While no single definitive model of a healthy and environmentally sustainable dietary pattern exists, more so in LMICs that are experiencing dietary transitions, it is generally agreed that such dietary practices revolve around a largely plant-based diet, with low to minimal animal-based products, including fish and poultry [5, 6, 14]. However, in order to put policies and interventions in place that encourage such dietary practices, it is imperative to first obtain an understanding of what dietary practices currently exist and what factors influence them. The aim of this study, therefore, was to identify multi-level factors in the lived rural and urban Ugandan food environments that influence existing dietary practices among women of reproductive age (WRA). WRA are of interest as they have reported poor outcomes for both over and under-nutrition in Uganda compared with older women and men of the same age-group [15].

## Methods

### Study setting and population

A cross-sectional, mixed method study design was used to address the aim of the project: a quantitative component established prevailing dietary practices, followed by a qualitative component that identified factors in lived rural and urban food environments that influence these dietary practices. Study participants were women aged 15–49 yrs. Urban participants were recruited from Nakawa division in Kampala district, the capital and largest urban settlement in Uganda. Rural participants were recruited from Nakawuka and Bulwanyi parishes in Wakiso district. Kampala and Wakiso districts were purposively chosen for pragmatic reasons, i.e. physical access and ease of communication (language). Gatekeepers facilitated participant recruitment across both study sites as recommended in a previous Photovoice study in Uganda [16]. The two gatekeepers in Wakiso were community health mobilisers, whereas in Kampala, one was a youth leader and the other a local women’s community leader. For the quantitative component of the study, which was carried out to establish existing dietary practices among WRA, participants were sampled using a quota sampling method. Quota sampling was used to ensure that a diversity of participants were populated into a priori groups based on SES and age. Age was divided into three categories, i.e. adolescents (15-19y), early adulthood (20-34y) and mid-adulthood (≥35y), while socioeconomic status (SES) was categorised into low, mid and high (based on the EquityTool for Uganda) [17]. The Equity Tool is based on asset ownership and represents individuals’ relative wealth compared with others in the same urban or national population [17]. The target sample size, comprising 3X age groups and 3X SES in either study site, was *n* = 54 (*n* = 27 urban; *n* = 27 rural) (Table S1). This ensured diversity in perspectives in the subsequent Photovoice that was carried out to assess factors influencing dietary practices established from the quantitative component. From the larger sample of participants that took part in the quantitative component, a smaller sample (*n* = 18; *n* = 9 urban and *n* = 9 rural) was randomly drawn to take part in the subsequent qualitative study using Photovoice and in-depth interviews. To achieve this, within each quota, ID numbers for all participants expressing interest in the qualitative component were written on individual pieces of paper, the papers folded and placed in a hat. One folded paper was picked from the hat and that participant’s ID was selected to represent that quota. This exercise was performed for each of the 18 quotas (a priori groups) from the quantitative component, until all quotas across both rural (*n* = 9) and urban (*n* = 9) study sites had one representative for the qualitative component of the study. For quotas (a priori groups) where only one participant expressed interest in the qualitative component, this participant represented that quota.

### Data collection tools

For the quantitative component, a paper-based questionnaire captured data on socio-demographic characteristics, dietary intake in the previous 24 h and the context of eating events. Dietary intake data were collected using the qualitative 24 h recall method [18]. Participants were asked to describe all food and drink consumed inside or outside the home on the day before the interview. However, unlike the traditional quantitative 24 h recall, participants did not estimate quantities consumed [18]. To prompt recall, a modified multi-pass method was used [19]. To this end, participants first listed all food and drink consumed the previous day from when they woke up until just before they slept [19]. Then, participants provided detailed descriptions of each item listed, specifying food preparation methods, such as boiled, fried or deep-fried beef. Next, participants answered follow-up questions on aspects surrounding each eating event, including length of eating event, when eating event took place and circumstances surrounding eating events. Lastly, for accuracy and completeness, the participants and interviewer reviewed the dietary recall [19]. At this point, participants were asked if there was any food/drink consumed between main meals that they might have forgotten. This was particularly important in Wakiso, where many participants omitted ‘snacks’ during the dietary recall because they were not regarded as ‘proper’ food in that context. At the end of each interview participants were also asked if the recall was reflective of their usual dietary behaviours (intakes, timing, etc.). Interviews lasted between 20 and 90 min and were conducted by the lead researcher (CIA). Field assistants (FAs) translated when necessary.

For the qualitative component, a modified Photovoice protocol [20] was used. First, a photography guide was prepared, containing five topic areas around which participants were required to take photographs, i.e. what is food, what does food mean to you, who do you eat with, where do you usually eat and how do you prepare your food. Then, participants were trained in Photovoice (its aims in the project, ethics of photography, photography skills and photography guide) by the lead researcher (CIA). Participants captured photographs over a one-week period, half-way through which they were contacted to discuss any challenges. Although participants were required to capture five photographs reflecting the five topic areas in the photography guide, participants were allowed additional photographs if they believed these more comprehensively illustrated their photo-stories. Following this activity, participants discussed their selected photographs with the lead researcher at in-depth interviews, lasting 30–120 min. In-depth interviews were administered using a paper-based interview guide based on the modified PHOTO technique [21], which framed discussion of participant photographs. The interview guide comprised the following questions: could you talk about or describe your **P**hoto; what is **H**appening in your photograph; why did you take a photograph **O**f this; what does this photo **T**ell us about food in your life; and how can this photo provide **O**pportunities for us to improve life. Of all participants sampled for the qualitative study (*n* = 18), some urban participants (*n* = 4) declined to take photographs, opting to only participate in interviews. The same interview guide was used for participants that did not take pictures, to ensure that all participants in the qualitative component answered similar questions. In these instances, participants were asked to imagine or reflect on what kind of photographs they would have taken if they had the cameras.

Data collection for this mixed methods study took place between July 2017 and January 2018. All data collection tools used in Kampala were prepared in English while those used in Wakiso were translated into *Luganda*, the local language by the lead researcher CIA. All translated material were double-checked by FAs for accuracy and corrections were made for ambiguities. The translated questionnaire was also piloted in Wakiso prior to data collection commencing. Interviews in Kampala were mainly conducted in English, while in Wakiso, both English and *Luganda* were used. The interviews in *Luganda* were carried out by CIA, who is knowledgeable in the language. However, FAs were available to provide nuance or context to participant narratives and translate interviews in instances where participants responses were unclear. All interviews were audio recorded. Interviews in Wakiso were conducted at a community health workers’ project office in Nakawuka parish while those in Kampala were conducted at a local church, with a few interviews conducted at home/workplaces at participants’ convenience.

### Data analysis

#### Dietary practices

Dietary typologies were generated using a two-step process. First, multiple correspondence analysis (MCA) was applied to the dietary data collected among the larger sample of participants that took part in the quantitative component of the study. MCA, a multi-variate data-reduction method, was used to group foods because the dietary data was in the form of a binary variable, i.e. consumed/not consumed, and not in quantifiable terms [22–24]. The MCA was run on 15 food groups (Table S2), which were formed based on various criteria [22–25], i.e. conventional food groups in the literature, reported frequency of consumption of the resulting food groups among study participants, environmental impact of constituent foods per 100 g and knowledge of the local context. Breaks in the scree plot, cumulative inertia > 40% and interpretability informed the decision on how many MCA dimensions to retain [26].

The first three MCA dimensions retained were then used as input variables to generate clusters using hierarchical cluster analysis (HCA) using the Ward’s criterion [26]. The interpretability of the partition (dendrogram) and agglomeration schedule were used to decide which dietary clusters to retain [26]. The stability of the four retained dietary clusters was tested using the split-half method [26, 27].

Following this, the four dietary clusters of participants’ dietary behaviours were labelled as ‘dietary typologies’. Labelling was based on location of participants constituting the dietary cluster (rural/urban), whether the food groups consumed by participants in the cluster were reflective of traditional Ugandan cuisine or a ‘modern’ diet and the environmental impact label of the food groups in the cluster, i.e. low, medium, high. The environmental impact label for each MCA food group was based on an environmental impact assessment exercise carried out in an unpublished study by the same author (Table S2). Environmental impact categories for food groups were obtained by first ranking environmental impact for all food groups in ascending order and then dividing this into tertiles. The food groups comprising the lowest tertile were classified as low-impact while those comprising the highest tertile were labelled high-impact. Those between high and low tertile were categorised as medium impact. Most participants mentioned that their dietary recalls were reflective of the foods they usually ate, except for some weekends and holidays like Christmas, etc. Therefore, the dietary typologies generated were taken as a proxy for participants’ usual dietary practices, although this was based on a one-day recall.

MCA and HCA were performed using Statistical Package for the Social Sciences (SPSS) Version 23, while the two-dimensional MCA bi plots were generated using GGplots function in RStudio.

#### Photovoice

For the qualitative data, thematic framework analysis was used because it is not attached to a specific theoretical discipline and is therefore widely adaptable, allows for the thematic comparison of participants’ accounts, and follows a systematic process, thereby providing an avenue for study improving validity and reliability [28]. All interviews were transcribed verbatim (translation of interviews conducted in *Luganda* occurred initially during interviews and then during transcription). Then, interview transcripts, supported by field notes, were checked for familiarisation and accuracy by CIA. Codes were mainly generated deductively using the socioecological model of factors influencing healthy eating behaviours [29]. However, coding was flexible hence allowing for the removal of irrelevant codes from the codebook or addition of codes arising from the data itself. Coding was first performed manually on paper with highlighter pens with a sample of transcripts (*n* = 3 rural and *n* = 3 urban) to generate a thematic framework. Coding for manually coded transcripts (*n* = 6) was later replicated in Nvivo. The thematic framework was then applied to the remaining interview transcripts (*n* = 12), themes generated and links and patterns between themes identified [29, 30]. Data validity and credibility were ensured by: i. recruiting a diverse quota sample, ii. relaying their narratives to each participant during the interviews to check for correctness, iii. using the thematic framework analysis method and iv. providing detailed descriptions of study methodology to allow a reader to make their own judgement on robustness [28, 31–35]. Management and analysis for qualitative data were performed using Nvivo Version 12.

## Results

### Socio-demographic characteristics of dietary clusters

The socio-demographic profile of study participants (*n* = 73) is summarised in Table 1. Most participants across the four dietary clusters were single and educated to at least the primary level (Table 1). Cluster 1 was the largest and youngest dietary cluster with an almost equal distribution of participants across the three SES levels (Table 1). Cluster 2 had a slightly higher proportion of rural than urban participants and half the cluster membership were of high SES (Table 1). Although Cluster 3 was the smallest dietary cluster, it had a significantly higher proportion of urban participants and participants at the highest SES level (*p* < 0.05) (Table 1). Cluster 3 was also more highly educated than all the other clusters (Table 1). Cluster 4 was characterised by largely rural, low SES participants (Table 1).

**Table 1:** Sociodemographic Characteristics of Dietary Clusters among Rural and Urban Ugandan WRA (*n* = 73)

	Cluster 1 ***n*** = 23 (31.5%)	Cluster 2 ***n*** = 22 (30.1%)	Cluster 3 ***n*** = 13 (17.8%)	Cluster 4 ***n*** = 15 (20.5%)	***p***-value
**Age,** yr (mean ± SD)	24.7 ± 8.7	28.0 ± 11.9	27.8 ± 8.7	29.0 ± 12.1	0.75
**Residence**					
Urban	16^a,b^ (69.6)	10^a,b^ (45.5)	10^b^ (76.9)	4^a^ (26.7)	0.02*
Rural	7^a,b^ (30.4)	12^a,b^ (54.5)	3^b^ (23.1)	11^a^ (73.3)	
**SES**					
Low SES	7^a^ (30.4)	7^a^ (31.8)	1^a^ (7.7)	6^a^ (40.0)	
Mid SES	7^a^ (30.4)	4^a^ (18.2)	2^a^ (15.4)	5^a^ (33.3)	0.21
High SES	9^a,b^ (39.1)	11^a,b^ (50.0)	10^b^ (76.9)	4^a^ (26.7)	
**Education**					
Less than primary	5^a^ (21.7)	6^a^ (27.3)	0^a^ (0.0)	3^a^ (20.0)	
Primary	15^a^ (65.2)	15^a^ (68.2)	10^a^ (76.9)	11^a^ (73.3)	0.50
Secondary	1^a^ (4.4)	0^a^ (0.0)	1^a^ (7.7)	0^a^ (0.0)	
Post-secondary	2^a^ (8.7)	1^a^ (4.5)	2^a^ (15.4)	1^a^ (6.7)	
**Marital Status**					
Single	15^a^ (65.2)	16^a^ (72.7)	8^a^ (61.5)	9^a^ (60.0)	0.85
Married	8^a^ (34.8)	6^a^ (27.3)	5^a^ (38.5)	6^a^ (40.0)	

*significant at 95% confidence level

^a, b, c^ values with different superscripts are significantly differ

### Dietary intake among dietary clusters

**Fig. 1:**
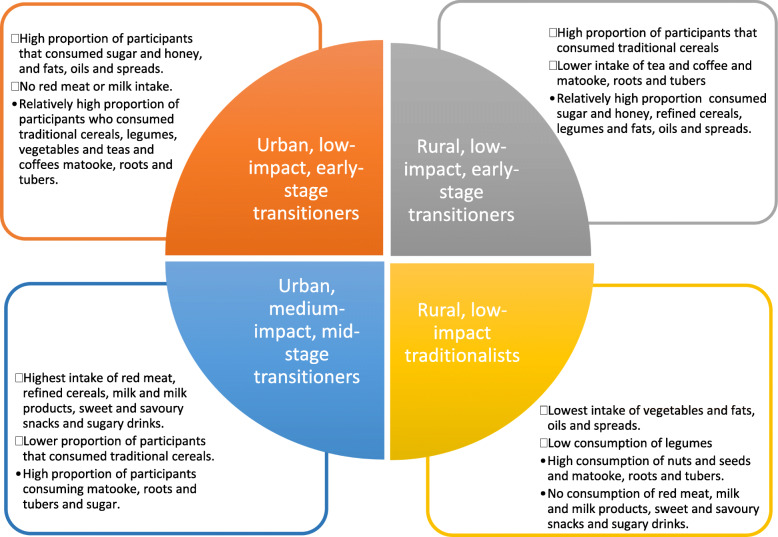
Reported Food Group Intake for Ugandan WRA in Four Dietary Typologies of Dietary Behaviour

Figure 1 summarises intake of food groups across the four dietary clusters among this sample of WRA (for further details see Table S3). Cluster 1 was characterised by participants that did not consume any high environmental impact animal foods (Fig. 1). Cluster 1 had a higher proportion of participants that consumed food groups associated with early-stage dietary transition in LMICs (sugar and honey and fats oils and spreads) [36]. This cluster also had a high proportion of participants that consumed diverse low environmental impact food groups (traditional cereals, legumes, vegetables and matooke, roots and tubers) (Fig. 1). Cluster 1 was labelled the ‘*urban, low-impact, early-stage transitioners*’ dietary typology. Cluster 2 was characterised by a higher proportion of participants that consumed traditional cereals and a lower proportion of participants that consumed low environmental impact teas and coffee and matooke, roots and tubers (Fig. 1). This dietary cluster also had a moderately high proportion of participants consuming refined cereals, legumes, fats, oils and spreads, sweet and savoury snacks and sugar and honey (Fig. 1). Because this dietary cluster was characterised by low vegetable intake and relatively high proportion of participants consuming food groups associated with early-stage dietary transition, this cluster was labelled the ‘*rural, low-impact, early-stage transitioners*’ dietary typology.

Cluster 3 was characterised by a lower proportion of participants that consumed traditional cereals and legumes (Fig. 1). This dietary cluster was also characterised by a higher proportion of participants that consumed medium and high environmental impact food groups (red meat, milk and milk products, refined cereals, sweet and savoury snacks and sugary drinks), which are indicative of a more advanced stage in dietary transition [36, 37]. Cluster 3 was labelled the ‘*urban, medium-impact, mid-stage transitioners*’ dietary typology. Lastly, Cluster 4 was characterised by the lowest proportion of participants consuming fats, oils and spreads and vegetables (Fig. 1). Participants in this dietary cluster also reported no consumption of red meat, milk and milk products, snacks or sugary drinks (Fig. 1). This cluster was labelled the ‘*rural, low-impact, traditionalists*’ dietary typology because the food groups for which it has no intake are often associated dietary transition [36, 37].

### Dietary typologies among women participating in Photovoice and in-depth interviews

Table 2 highlights the cluster membership of participants who took part in Photovoice and in-depth interviews. Study participants were distributed equally (*n* = 5) across the ‘*urban, low-impact, early-stage transitioners*’, the ‘*rural, low-impact, early-stage transitioners*’ and the ‘*urban, medium-impact, mid-stage transitioners*’ (Table 2). The ‘*rural, low-impact traditionalists*’ dietary typology had the smallest cluster membership (Table 2). While rural participants were largely clustered into the ‘*rural, low-impact, early-stage transitioners*’ and the *rural, low-impact traditionalists*’ dietary typologies, all urban participants belonged either to the ‘*urban, low-impact, early-stage transitioners*’, the ‘*rural, low-impact, early-stage transitioners*’ or the ‘*urban, medium-impact, mid-stage transitioners*’ dietary typologies (Table 2). Most Photovoice participants in both the ‘*urban, medium-impact, mid-stage transitioners*’ and the ‘*rural, low-impact, early-stage transitioners*’ dietary typologies were of mid and high SES (Table 2).

**Table 2:** Dietary Typologies among WRA Participating in Photovoice and In-depth interviews (*n* = 18)

Participant no.	Age (yrs)	SES	Education Completed	Occupation	Marital Status	Location
‘Urban, medium-impact, mid-stage transitioners’ dietary typology (*n* = 5)						
Participant 1	33	High	Post-secondary	Lawyer	Single	Urban
Participant 2	22	Mid	Primary	Shop attendant	Single	Urban
Participant 3	42	High	Secondary	Businesswoman	Single	Urban
Participant 7	15	High	Primary	Student	Single	Urban
Participant 17	17	Low	Primary	Unemployed	Single	Rural
‘Urban, low-impact, early-stage transitioners’ dietary typology (*n* = 5)						
**Participant 6**	**25**	**Low**	**Primary**	**Housewife**	**Married**	**Urban**
Participant 8	17	Low	Not completed primary	Restaurant worker	Single	Urban
**Participant 9**	**17**	**Mid**	**Primary**	**Student**	**Single**	**Urban**
Participant 11	19	High	Secondary	Unemployed	Single	Rural
Participant 14	20	Low	Primary	Peasant farmer	Married	Rural
‘Rural, low-impact, early-stage transitioners’ dietary typology (*n* = 5)						
Participant 10	16	High	Not completed primary	Restaurant worker	Single	Rural
**Participant 4**	**44**	**Mid**	**Primary**	**Tailor**	**Married**	**Urban**
Participant 15	16	Mid	Primary	Unemployed	Single	Rural
Participant 16	30	Mid	Primary	Unemployed	Single	Rural
**Participant 5**	**42**	**Low**	**Secondary**	**Unemployed**	**Single**	**Urban**
‘Rural, low-impact, traditionalists’ dietary typology (*n* = 3)						
Participant 12	41	High	Primary	Peasant farmer	Married	Rural
Participant 13	39	Mid	Primary	Peasant farmer	Single	Rural
Participant 18	37	Low	Not completed primary	Contract farmer	Married	Rural

*Participants in bold declined to take photographs and only participated in the in-depth interviews (source Auma 2020)

### Factors influencing dietary practices

**Fig. 2:**
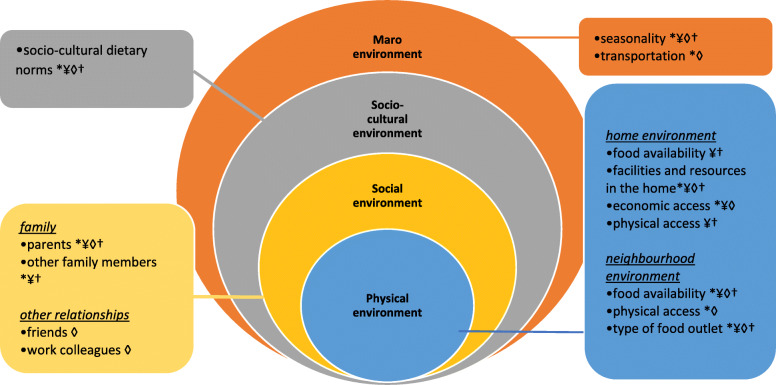
Factors influencing Healthy and Environmentally Sustainable Dietary Practices among Four Dietary Typologies of Ugandan WRA (adapted from Story et al. [29]) *urban, low-impact, early-stage transitioners; ¥rural, low-impact, early-stage transitioners, ◊urban, medium-impact, midstage. Transitioners, †rural, low-impact traditionalists

Participants in this study highlighted factors that influenced their dietary practices at various levels, i.e. social environment (familial and other relationships), physical environment (food access, food availability, type of neighbourhood food outlet and economic access), socio-cultural dietary norms and the macro-environment (seasonality and transport) (Fig. 2). Although the factors were commonly expressed by participants, they were sometimes experienced differently in the four dietary typologies (Fig. 2).

### Social environment

Photovoice participants spoke of the social environment influencing their dietary practices through the channel of family, and to a lesser extent friends and work colleagues. Family influence is highlighted in this section. Many WRA across all four dietary typologies strongly alluded to ‘*role modelling*’ as pertinent to how their parents influenced their dietary practices. Participants’ narratives highlighted that parental influence firstly established, and then over time, entrenched certain dietary practices from childhood to adulthood. Most younger participants from the ‘*urban, medium-impact, mid-stage transitioners*’ dietary typology, for example, spoke about this in terms of provisioning, i.e. what was made available. Since they were still in their parents’ care, these participants expressed that they felt they often had no choice but to eat what was provided, primarily by their mothers. On the other hand, some younger participants in this dietary typology spoke of fathers refusing to eat certain things, particularly fried foods, and therefore imposing their food preferences over the entire household. Similar sentiments were shared by many similarly aged ‘*rural, low-impact, early-stage transitioners*’ dietary typology participants. These collective narratives among younger participants illustrated gender dynamics in the parental influence around dietary practices; while mothers appear to play a more direct role by limiting what was available through provisioning, fathers act less directly by establishing an ‘unspoken law’ as one participant illustrates:

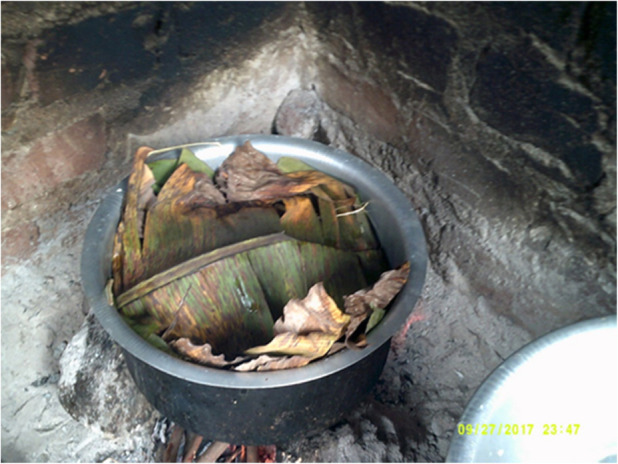

*‘If my dad however is going to eat the ‘nakatti’ [traditional vegetables], or any other thing for that matter, and he prefers for the food not to be fried, then all of us will eat boiled food that day.’ (Participant 17, ‘urban, medium-impact, mid-stage transitioners’, rural, 15-17Y).*

For older ‘*urban, medium-impact, mid-stage transitioners*’ dietary typology participants, parental influence was less exacting. Mothers, rather than provisioning or ‘enforcing the father’s law’, advised participants, for example, to eat steamed/boiled rather than fried vegetables, but allowed them to make their own decisions. Beyond parental influence, participants in the ‘*urban, medium-impact, mid-stage transitioners*’ dietary typology spoke often of the influence of friends on their dietary practices, while the influence of ‘other non-parent family members’ was more pertinent among participants across the other three dietary typologies. In these instances, participants made a distinction between how eating in the company of their spouses and children, rather than by themselves, encouraged them to eat foods they disliked, such as white rice and potatoes, in place of lower environmental impact traditional staples like matooke.

### Physical environment

Key sub-themes that emerged in the physical food environment included type(s) of food available within the household and neighbourhood food outlets, physical access to neighbourhood food outlets and economic access (cost) of food at the neighbourhood food outlets. Participants across the four dietary typologies, particularly those from the two rural dietary typologies, spoke of eating certain healthier, lower environmental impact, plant-based foods because they were readily available within their households. This availability was in turn dictated by the presence (or absence) of different resources or facilities within the home environment. For many participants across the four dietary typologies, these resources or facilities included home gardens that allowed participants to grow and regularly eat fresh, plant foods, e.g. fruit, vegetables, legumes (beans and groundnuts), roots and tubers (sweet potatoes and cassava), matooke and some grains (maize). The dominance of perspectives on ‘home gardens’ by participants from the two rural dietary typologies suggests that participants in the two urban dietary typologies supplemented own production with other food sources while dietary practices of participants from the two rural typologies are more closely tied to ‘own production’ as illustrated below:

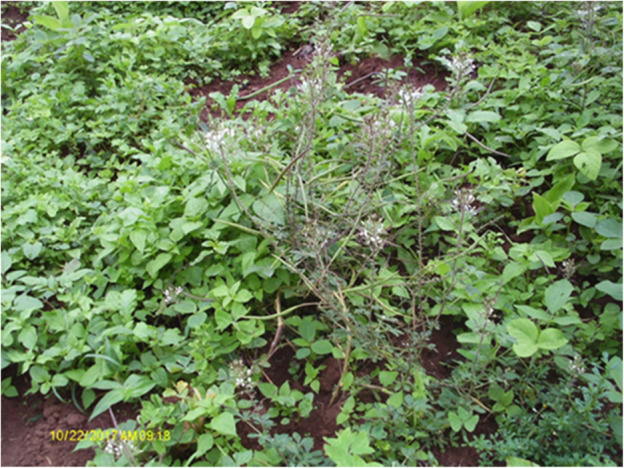

*‘…that is the garden from which we get our food.... Although you cannot see it properly, there is some cassava in there. What you can see clearly is the ‘gyobyo’ [spider plant], soya beans and beans. …there is another photo I took of [another section of] the garden, showing maize and groundnuts...’ (Participant 12, ‘rural, low-impact, traditionalist’*, *rural, 35-49Y).*

Different from home gardens, ‘*urban, medium-impact, mid-stage transitioners*’ dietary typology participants, highlighted the role of other facilities, e.g. ovens and refrigerators. In addition to enabling participants to cook and make available different foods, these resources facilitated the storage of food so that participants could eat them whenever they wanted. One urban participant’s narrative demonstrated why she regularly ate ‘githeri minji’, a lower environmental impact, plant-based, traditional dish.

*‘Now, the surprising thing is this thing [githeri minji] has been in my fridge since September … the mixture of maize and peas since September. So, I boiled it and I froze it. So, I have been having it in my fridge.’ (Participant 1, ‘urban, medium-impact, mid-stage transitioners’, urban, 18–34Y).*

While the home environment is clearly important in providing the foods required to prepare ‘githeri minji’ (maize and beans), the importance of the refrigerator is emphasised. By being the place in which this participant stores cooked ‘githeri minji’, the refrigerator provides an enabling environment that allows the participant to partake in the practice of eating this dish whenever the need arises. From these collective examples it can be observed that food availability within the home environment is mediated by participant-owned materials, e.g. home gardens and some electronic appliances, which collectively create an enabling environment that supports the enactment of some healthier and lower environmental impact dietary practices that revolve round the consumption of mostly plant foods among these WRA.

Furthermore, within the physical environment, participants in three dietary typologies (‘*urban, low-impact, early-stage transitioners*’, ‘*rural, low-impact, early-stage transitioners*’ and ‘*urban, medium-impact, mid-stage transitioners*’) highlighted high food prices as influencing their dietary practices. Finances were pivotal to decision-making regarding places from where participants shopped and whether they could afford to purchase certain food. This often explained why they bought certain foods over others. For ‘*urban, medium-impact, mid-stage transitioners*’, cost was a barrier to less healthy dietary practices associated with more advanced stages in dietary transitions, i.e. regularly eating out at high-end restaurants and purchasing sugar-sweetened beverages. Therefore, for many participants in this dietary typology, cost of food is often presented as a barrier towards the consumption of ‘transitioning’ foods that might be considered ‘luxurious’ or ‘modern’. On the other hand, for ‘*urban, low-impact, early-stage transitioners*’ and ‘*rural, low-impact, early-stage transitioners*’ participants concerns about food cost limited the purchase of even the most basic foods required daily. Participants in these two dietary typologies found cost especially limiting when healthier, lower environmental impact fruit, vegetables and legumes were out of season. Furthermore, participants in these two dietary typologies emphasised the high cost of higher environmental impact, animal-based foods, so much so that they were forced to consume these products irregularly, reserving them only for what they described as ‘*big days*’, e.g. Christmas, New Year. For these participants, cost served as a deterrent to regular meat consumption.

When food cost presented less of a barrier, participants across all dietary typologies demonstrated that the kinds of food available to them in neighbourhood food outlets could either be a limitation or an enabler to certain ways of eating. Neighbourhood food availability was particularly important for *‘urban, low-impact, early-stage transitioners*’, ‘*rural, low-impact, early-stage transitioners*’ and *‘urban, medium-impact, mid-stage transitioners’*, and less so among ‘*rural, low-impact traditionalists*’ participants as one participant showed:

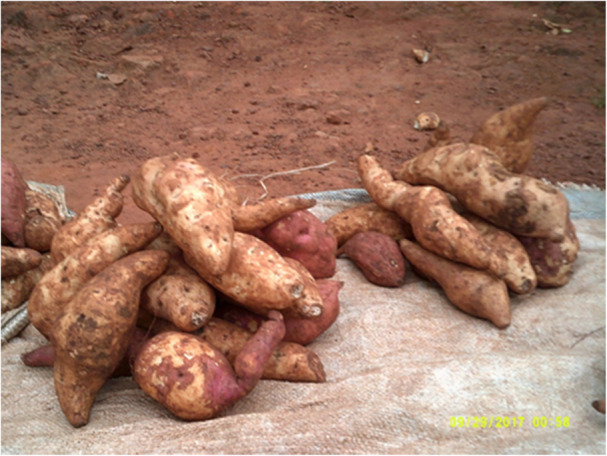

*‘Sweet potatoes are the food that are readily available and nearest to us, and so they are the food we usually eat. Moreover, at that stall from which we buy food, it is what is available. At that stall, they do not sell anything else like rice. All she has is sweet potatoes and matooke’ (Participant 17, ‘urban, medium-impact, mid-stage transitioners, rural, 15-17Y).*

Lastly, for participants that sourced food from beyond their home gardens, physical access to neighbourhood food outlets influenced dietary practices. Since most participants in the two rural dietary typologies spoke of largely producing their own food, it was unsurprising that physical access was more salient among participants in the two urban dietary typologies. For these participants in the two urban dietary typologies, proximity to neighbourhood restaurants, supermarkets and shops provided convenience, especially when they were pressed for time. In these circumstances, food choice decisions that were previously enacted in similar time-limited circumstances, came to the fore resulting in participants eating convenience foods in place of home-cooked meals, for example.

### Socio-cultural factors

Many participants across the four dietary typologies spoke of how the sociocultural food contexts in which they had previously lived influenced their dietary practices. In this regard, participants across all four dietary typologies used terms such as ‘the way we grew up’, ‘our food’ and ‘in our culture’ to denote attachment to their traditional foods and traditional ways of doing things, for example, steaming food in banana leaves rather than frying. In the instances where participants spoke of ties to their cultural heritage, their upbringing was often when they reported encountering these traditions or dietary norms. ‘*Rural, low-impact traditionalists*’, for example, seemed generally resolute that, based on these dietary norms, it was best to eat plenty of steamed vegetables. Among these participants, the frying of vegetables carried a negative connotation, which was learned from their parents, i.e. the addition of vegetable oil to traditional vegetables somehow made them less healthy (‘bad’). This same thinking explained why some ‘*rural, low-impact, early-stage transitioners*’ and ‘*rural, low-impact, traditionalists*’ participants spoke of eating meat less frequently (or avoiding it completely) compared with participants in the two urban dietary typologies. Participants’ accounts illustrated that eating (or not eating) certain foods in adulthood has a great deal of meaning beyond merely eating. It is an act of paying homage to the culture in which they were raised, i.e. old ways of doing things. While most participants reported maintaining these childhood-established dietary norms in adulthood, two ‘*urban, low-impact, early-stage transitioners*’ participants reported otherwise. Participant 6, an unemployed 25-year-old housewife who lived with her family in a low-income informal urban settlement spoke of deliberately seeking out things that were non-normative of her childhood diet and avoiding those foods she felt she had too much of as a child. From this participant’s narratives, becoming an adult and achieving autonomy over her home (and therefore food choices), as well as relocating from the village to the city exposed her to a wider diversity of foods, which facilitated a deviation from her ‘traditional’ dietary norms. On the other hand, Participant 14, a married 20-year-old peasant farmer that lived in rural Bulwanyi with her family, spoke of moving into her husband’s home as forcing her to substitute her childhood norms of dietary practice (centred around medium environmental impact ‘matooke’) with cassava and sweet potatoes (lower environmental impact roots and tubers) because that was the norm in her new family.

### Macro-environment factors

While participants across all four dietary typologies spoke less of macro-level influences, some evoked the role of seasonal food production. Seasonality was reported to directly influence dietary practices by dictating what foods could grow at different periods of the year, and therefore, availability. The macro environment seemed particularly important to participants in the two rural dietary typologies given that most of them spoke of cultivating the bulk of what they ate from their home gardens. Among participants in the two urban dietary typologies, seasonality influenced dietary practices in a slightly different form. When certain foods were in season in other parts of the country, it provided a buffer against high prices in the urban markets. This, coupled with the transport infrastructure from rural areas where most foods are produced, meant that such urban participants could have better economic access to plant foods in season. Furthermore, while the transport infrastructure serves as an enabling factor in the consumption of some (dried) foods among urban dietary typology participants, it is in equal measure a disabling factor in the consumption of healthier, lower environmental impact perishable foods in-season. Conclusively, participants’ narratives indicated that when it comes to healthier, lower-environmental impact plant foods, while seasonality plays a direct role among participants in the two rural dietary typologies, seasonality appears to play a more distal role among urban dietary typology participants.

## Discussion

The aim of this cross-sectional study was to identify multi-level factors in the lived rural and urban Ugandan food environments that influence existing dietary practices among a sample of WRA. Four dietary typologies emerged, i.e. ‘*urban, low-impact, early-stage transitioners*’, ‘*urban, medium-impact, mid-stage transitioners*’, ‘*rural, low-impact, early-stage transitioners*’ and ‘*rural, low-impact, traditionalists’*. Although expressed somewhat differently, participants across all four dietary typologies highlighted the physical environment (food access, availability and cost), social networks (parents, other family members and friends) and socio-cultural dietary norms as important. Seasonality and transportation (macro-environment) influenced the consumption of healthier, lower environmental impact, plant-based foods among participants from the two urban dietary typologies.

As highlighted, four dietary typologies emerged among this sample of rural and urban WRA, i.e. two rural and two urban dietary typologies. Of these, three dietary typologies, i.e. the ‘*urban, low-impact, early-stage transitioners*’, the ‘*urban, medium-impact, mid-stage transitioners*’ and the ‘*rural, low impact, early-stage transitioners*’ were indicative of transitioning dietary practices among these WRA. The ‘*rural, low-impact, traditionalists*’, on the other hand, was more illustrative of traditional dietary practices. Overall, some similarities were observed between the two urban dietary typologies in this study and the ‘*urban*’ dietary pattern observed among Burkinabe adults [38], while differences were seen between the ‘*urban, medium-impact, mid-stage transitioners*’ dietary typology and the ‘*transitional*’ dietary patterns among West African immigrants in Madrid [39] as well as the ‘*unsustainable*’ dietary pattern among Irish adults [40]. On the other hand, the ‘*traditional*’ dietary cluster among Burkinabe adults [36] was strikingly like the ‘*rural, low-impact, early-stage transitioners*’ dietary typology, but not the more traditional ‘*rural, low-impact, traditionalists*’ dietary typology. Having both one traditional and three transitioning dietary typologies among the same sample of WRA is suggestive of a ‘traditional-transitional’ dietary gradient, which has been observed in other SSA contexts [41, 42] as well as studies from some middle-income countries experiencing more advanced stages of dietary transition, e.g. Mexico [43]. Important to note, however, is that this present study was conducted on a small sample of WRA therefore findings have limited generalisability to all Ugandan WRA. However, findings offer a starting point to understanding dietary typologies and factors surrounding dietary practice in a low-income, transitioning context. Furthermore, another strength of this study is its focus on both rural and urban participants compared with other studies which draw their sample from either urban or rural locations.

The influence of social networks on dietary practices across all four dietary typologies was important. Women spoke extensively of parents, family and friends, among others, influencing how they cooked and ate. Younger participants in our study spoke of parents either providing certain food items or establishing rules on what could be eaten within the household. While there is a dearth of literature on the social environment and dietary practices in SSA [44], a few studies have reported similar findings. Among rural and urban Cameroonian [45] and urban South African adolescents [46], for example, participants reported eating leafy green vegetables because their mothers made it available at home. Narratives from participants in our study also indicated that friends and peers influence dietary practices of younger women, particularly. Similar findings were reported among adult urban-poor Ghanaians, rural and urban Cameroonian adolescents and urban South African adolescents who all reported being motivated to eat energy-dense, nutrient-poor (EDNP) foods in the company of friends and peers [46, 47]. Indeed, some authors have highlighted that people often ‘mirroring’ the dietary practices of those within their social networks to impress them or signal belonging [48]. Older participants in this study also demonstrated that other (non-parent) family members were important influences around their dietary practices. These findings corroborate those from a recent study among Indian women, who reported that in trying to make them happy, they often ate food that they knew their spouses and children preferred [49]. Food in Uganda, as in many other SSA countries, is an identifier of cultural heritage and tradition. As such, food is deeply embedded in people’s daily lives. To mark important social and cultural events, e.g. marriages, births and deaths, special foods are often eaten [50]. This could shed light on why many women across the four dietary typologies spoke of eating ‘special foods’, e.g. meat, chicken, white rice, dairy, fried food on ‘*big days*’ of celebration. Such ‘special foods’, are characteristic of the dietary changes associated with the nutrition transition, as reported in a South African study [51].

Narratives among older participants in this study highlighted links between social networks and the socio-cultural environment resulting in many participants often making unconscious decisions regarding dietary practices. For example, participants across the four dietary typologies spoke of eating ‘little oil’ or not wanting ‘too much fats’ seemingly out of habit, i.e. this is what they had always done since their childhood and could not imagine doing otherwise. Eating practices (dietary norms) established during participants’ childhoods led to persistent patterns of food choice in adulthood. These habituations, while enforced by participants’ parents, were often a product of the socio-cultural environment in which participants were raised. The pivotal role of parents, particularly mothers, in shaping children’s dietary practices, whether as role-models or providers, is well-documented in various studies in both HICs and LMIC contexts. For example, a 2018 paper highlighted the role of socialisation by mothers, during participants’ childhood, in shaping the dietary practices of urban-poor Ghanaian men and women into adulthood [47]. However, while participants in this mixed methods study, like those in other SSA studies, largely spoke of maintaining childhood-established dietary norms, some urban participants in the ‘*rural, low-impact, early-stage transitioners*’ spoke of aspirations to include more meat and dairy in their diets if their finances improved. These findings corroborate those from a study among urban South Africans, in which participants who moved to urban townships, improved their SES and ate more meat and chicken, reporting a desire to eat foods they had been deprived of during childhood, as proof of their improved financial status [51]. However, in this study it is important to highlight that no participants spoke of completely replacing their traditional diets with ‘modern’ foods. This is like other SSA studies, in which both traditional and modern diets coexist, which is unsurprising given that dietary transition is a gradual process.

Participants highlighted food availability as a motivator towards consuming healthier, lower environmental impact fruit and vegetables, roots and tubers. While household availability seemed more pertinent among participants from the two rural dietary typologies because they largely produced their own food, participants in the two urban dietary typologies recognised neighbourhood food availability as more important. It has been argued that urban residents generally have more diverse food options than their rural counterparts owing to a wider variety of neighbourhood food sources, including shops, supermarkets, street food outlets, restaurants and markets [52]. However, many ‘*urban, medium-impact, mid-stage transitioners*’, ‘*urban, low-impact, early-stage transitioners*’ and ‘*rural, low-impact, early-stage transitioners*’ demonstrated that availability does not necessarily translate into the enactment of certain dietary practices. Participants demonstrated that physical and economic access were just as important in influencing their healthier, lower environmental impact dietary practices. Physical access was discussed by most participants in this study in terms of distance between their homes and various food sources. For participants who had home gardens, immediate access to them was particularly enabling in the enactment of dietary practices that involved the consumption of lower environmental impact, plant-based food groups, e.g. fruit, vegetables, legumes, matooke, roots and tubers. For participants that did not have home gardens, particularly urban women, nearness to neighbourhood food outlets was salient. Physical access often interacted with convenience, when participants were faced with time constraints. However, findings from this study demonstrate that physical access and availability of both healthy and unhealthy foods in urban areas do not necessarily imply consumption among urban residents. In addition to these two factors, the high cost of healthier, lower environmental impact foods, such as fruits, vegetables and healthier as well as higher environmental impact animal foods, compared with the lower cost of EDNP foods could explain higher meat and dairy consumption among the ‘*urban, medium-impact, mid-stage transitioners’* (urban-rich) relative to the ‘*urban, low-impact, early-stage transitioners*’ (urban-poor). The role of food price in influencing dietary practices is demonstrated by many studies in SSA, for example fruit and vegetables were considered expensive and thus consumption was limited among urban-poor Ghanaians [47], while urban female South African adolescents and rural male and female Cameroonian adolescents considered EDNP convenience foods more affordable than healthier options [45, 46]. In summary, findings from this study on the physical environment collectively corroborate what has previously been proposed that while income shapes economic access to food, physical access shapes what is available for purchase [52]. It is almost impossible, therefore, to consider the influence on dietary practices of these three factors in isolation.

Lastly, on a macro level, findings from this study appear to contradict the narrative that urban residents in LMICs necessarily have greater access to marketing and are therefore more inclined to purchase EDNP foods, which comprise the bulk of food adverts [52, 53]. Participants in this study hardly spoke of the media, advertising or product branding influencing how they made food-purchasing decisions or what they ate. This does not mean to say that advertising was absent in the two study contexts, more so in urban Kampala. Participants, particularly those from the two rural dietary typologies, spoke extensively about seasonality being a major macro-environment level influence over their consumption of healthier, lower environmental impact plant-based food groups, most notably fruits and vegetables. Previous studies demonstrate that in SSA, fruit and vegetables intake, especially by rural populations that largely produce their own food, is highly season dependent. As such, fruits and vegetables are consumed in abundance when they are in season and hardly consumed when they are out of season [53–55]. On the other hand, participants from the two urban dietary typologies hardly mentioned food seasonality directly influencing their healthier, lower environmental impact dietary practices, although it was alluded to when some participants spoke of variations in food cost through the year. This could be because urban residents, especially the ‘urban-rich’, generally have access to both increased income and a wider variety of foods, sourced from food-producing rural areas, including refrigerated and frozen options in supermarkets [41, 53, 54]. These act as buffers, among the urban-rich against variation between seasons. On the other hand, the ‘urban-poor’ might have challenges accessing fruits and vegetables at different times of the year as they become more expensive out-of-season relative to when they are in season.

While this study provides a description of healthy and environmentally sustainable dietary practices among this sample of WRA, the use of a single qualitative 24 h recall among a relatively small sample (*n* = 73) means that these findings are not necessarily indicative of habitual intake. Environmental impact values used in the study are only estimates. This study was of cross-sectional; therefore, dietary typologies are likely to change with time. Dietary practices in this paper only relate to this sample of WRA and have limited generalisability to all rural and urban Ugandan WRA. Lastly, not all urban women took photographs in the Photovoice exercise for various reasons. However, the fact that the same interview guide was used for all in-depth interviews, to some extent, mitigated this.

## Conclusion

Participants displayed a range of dietary typologies, and therefore dietary practice. Family provides an avenue through which interventions aimed at encouraging healthier and lower environmental impact dietary practices can be targeted among both rural and urban WRA. Interventions and policies promoting the use of home gardens, urban farming and improved transportation could address challenges in availability and access to healthier, lower environmental impact plant-based foods among urban WRA, especially the urban-poor.

## Supplementary information

**Additional file 1: Table S1.** Sampling Matrix for Study Participants Showing Expected and Actual Sample Size. **Table S2.** Nutrient Density and Environmental Impact Categories for MCA Food Groups. **Table S3.** Consumption of Food Groups among Dietary Clusters of Rural and Urban Ugandan WRA (*n* = 73).

## Data Availability

The datasets used during the current study, except for Photovoice-generated participant photographs, are available from the corresponding author on reasonable request.

## Abbreviations

HCA: Hierarchical Cluster Analysis
HICs: High-income countries
EDNP: Energy-dense, nutrient-poor
LMICs: Low-income countries
MCA: Multiple Correspondence Analysis
NR-NCDs: Nutrition-related non-communicable diseases
SES: Socioeconomic status
SSA: Sub-Saharan Africa
WRA: Women of reproductive age
