# Supplementary material for: Factors influencing dietary practices in a transitioning food environment: a cross-sectional exploration of four dietary typologies among rural and urban Ugandan women using Photovoice
Source: Nutr J. 2020 Nov 25;19:127. doi: 10.1186/s12937-020-00634-9 (PMC7690007; doi:10.1186/s12937-020-00634-9)
Supplement: Supplementary file 1 — Additional file 1: Table S1. Sampling Matrix for Study Participants Showing Expected and Actual Sample Size. Table S2. Nutrient Density and Environmental Impact Categories for MCA Food Groups. Table S3. Consumption of Food Groups among Dietary Clusters of Rural and Urban Ugandan WRA (n = 73). [file 12937_2020_634_MOESM1_ESM.docx]

Supplementary Table 1: Sampling Matrix for Study Participants Showing Expected and Actual Sample Size

| SES  Age group | Low SES | | Mid SES | | High SES | | n |
| --- | --- | --- | --- | --- | --- | --- | --- |
| 15-19y (Adolescence) | Rural | Urban | Rural | Urban | Rural | Urban | 18  (24) |
|  | 3  (n=3) | 3  (n=7) | 3  (n=3) | 3  (n=3) | 3  (n=3) | 3  (n=5) |  |
| 20-34y (Early adulthood) | 3  (n=3) | 3  (n=3) | 3  (n=3) | 3  (n=4) | 3  (n=7) | 3  (n=8) | 18  (28) |
| 35-49y  (Mid adulthood) | 3  (n=3) | 3  (n=3) | 3  (n=3) | 3  (n=3) | 3  (n=5) | 3  (n=4) | 18  (21) |

Final quota sample size for participants recruited to the study indicated in brackets (source Auma 2020)

Supplementary Table 2: Nutrient Density and Environmental Impact Categories for MCA Food Groups

| **Food Groups** |  | **NRF11.3_100g_** | **Nutrient density category** | **GHGE_100g_** | **Environmental impact category** |
| --- | --- | --- | --- | --- | --- |
| Red meat |  | 57.9 | Medium | 813.4 | High |
| Poultry, fish and eggs |  | 89.5 | High | 832.1 | High |
| Milk and milk products |  | 9.4 | Low | 88.5 | Medium |
| Traditional cereals |  | 25.9 | Low | 11.0 | Low |
| Non-traditional cereals |  | 36.8 | Medium | 70.5 | Medium |
| Matooke, roots and tubers^^[[1]](#footnote-1)^^ |  | 46.8 | Medium | 26.7 | Low |
| Legumes |  | 80.9 | High | 25.8 | Low |
| Nuts and seeds |  | 132.3 | High | 47.9 | Medium |
| Fruit |  | 51.7 | Medium | 64.7 | Medium |
| Vegetables |  | 104.6 | High | 38.3 | Low |
| Fats, oils and spreads |  | 7.3 | Low | 376.5 | High |
| Sugary drinks^^[[2]](#footnote-2)^^ |  | 11.6 | Low | 28.3 | Low |
| Sweet and savoury snacks |  | 4.3 | Low | 129.3 | High |
| Sugar and honey |  | -69.6 | Low | 6.0 | Low |

Nutrient density and environmental impact categories were determined by dividing nutrient density scores, generated using the NRF11.3 nutrient profiling model into tertiles. Nutrient densities for each food group were first generated by compiling the weighted average of all food items comprising each food group. Then nutrient densities for food groups were ranked in ascending order, and these divided into tertiles. The food groups in the lowest tertile were labelled as ‘low’ nutrient density, those in the highest tertile were ‘high’ nutrient density foods and those in the middle were labelled as ‘medium’ nutrient density foods. A similar exercise was performed for environmental impact categories using previously published greenhouse gas emission data.

Supplementary Table 3: Consumption of Food Groups among Dietary Clusters of Rural and Urban Ugandan WRA (n=73)

|  | **Cluster 1**  **n=23 (31.5%)** | **Cluster 2**  **n=22 (30.1%)** | **Cluster 3**  **n=13 (17.8%)** | **Cluster 4**  **n=15 (20.5%)** | **p-value** |
| --- | --- | --- | --- | --- | --- |
| Red meat | 0^a^ (0) | 1^a^ (4.5) | 9^b^ (69.2) | 0^a^ (0) | 0.00 |
| Poultry and fish | 2^a^ (8.7) | 4^a^ (18.2) | 6^a^ (46.2) | 2^a^ (13.3) | 0.06 |
| Milk and milk products | 0^a^ (0) | 4^a,b^ (18.2) | 4^b^ (30.8) | 0^a,b^ (0) | 0.01 |
| Traditional cereals | 18^a,b^ (78.3) | 21^b^ (95.5) | 3^c^ (23.1) | 7^a,c^ (46.7) | 0.00 |
| Refined cereals | 11^a^ (47.8) | 11^a^ (50.0) | 13^b^ (100) | 2^a^ (13.3) | 0.00 |
| Matooke, roots and tubers | 20^a^ (87.0) | 8^b^ (36.4) | 11^a^ (84.6) | 15^a^ (100) | 0.00 |
| Legumes | 20^a^ (87.0) | 12^a,b^ (54.5) | 1^c^ (7.7) | 5^b,c^ (33.3) | 0.00 |
| Nuts and seeds | 5^a^ (21.7) | 2^a^ (9.1) | 5^a^ (38.5) | 7^a^ (46.7) | 0.045 |
| Fats, oils and spreads | 23^a^ (100) | 15^b^ (68.2) | 9^b^ (69.2) | 1^c^ (6.7) | 0.00 |
| Fruit | 3^a^ (13.0) | 3^a^ (13.6) | 6^a^ (46.2) | 2^a^ (13.3) | 0.09 |
| Vegetables | 23^a^ (100) | 22^a^ (100) | 13^a,b^ (100) | 10^b^ (66.7) | 0.00 |
| Sugar and honey | 23^a^ (100) | 12^b^(54.5) | 9^b^ (69.2) | 8^b^ (53.3) | 0.00 |
| Sweet and savoury snacks | 2^a,b^ (8.7) | 4^a,b^ (18.2) | 5^b^ (38.5) | 0^a^ (0) | 0.03 |
| Sugary drinks | 1^a^ (4.3) | 1^a^ (4.5) | 7^b^ (53.8) | 0^a^ (0) | 0.00 |
| Tea and coffee | 23^a^(100) | 6^b^ (27.3) | 10^a^ (76.9) | 13^a^ (86.7) | 0.00 |

1. includes katogo [↑](#footnote-ref-1)
2. includes sweetened teas, sweetened coffee and sweetened fruit juices and drinks [↑](#footnote-ref-2)
